# Learning protein binding affinity using privileged information

**DOI:** 10.1186/s12859-018-2448-z

**Published:** 2018-11-15

**Authors:** Wajid Arshad Abbasi, Amina Asif, Asa Ben-Hur, Fayyaz ul Amir Afsar Minhas

**Affiliations:** 1 Biomedical Informatics Research Laboratory (BIRL), Department of Computer and Information Sciences (DCIS), Pakistan Institute of Engineering and Applied Sciences (PIEAS), Nilore, ISL 45650 Pakistan; 2 Information Technology Center (ITC), University of Azad Jammu & Kashmir, Muzaffarabad, Azad Kashmir 13100 Pakistan; 3 Department of Computer Science, Colorado State University (CSU), Fort Collins, CO 80523 USA

**Keywords:** Protein-protein interactions, Protein binding affinity prediction, Privileged information, Machine learning

## Abstract

**Background:**

Determining protein-protein interactions and their binding affinity are important in understanding cellular biological processes, discovery and design of novel therapeutics, protein engineering, and mutagenesis studies. Due to the time and effort required in wet lab experiments, computational prediction of binding affinity from sequence or structure is an important area of research. Structure-based methods, though more accurate than sequence-based techniques, are limited in their applicability due to limited availability of protein structure data.

**Results:**

In this study, we propose a novel machine learning method for predicting binding affinity that uses protein 3D structure as privileged information at training time while expecting only protein sequence information during testing. Using the method, which is based on the framework of learning using privileged information (LUPI), we have achieved improved performance over corresponding sequence-based binding affinity prediction methods that do not have access to privileged information during training. Our experiments show that with the proposed framework which uses structure only during training, it is possible to achieve classification performance comparable to that which is obtained using structure-based features. Evaluation on an independent test set shows improved performance over the PPA-Pred2 method as well.

**Conclusions:**

The proposed method outperforms several baseline learners and a state-of-the-art binding affinity predictor not only in cross-validation, but also on an additional validation dataset, demonstrating the utility of the LUPI framework for problems that would benefit from classification using structure-based features. The implementation of LUPI developed for this work is expected to be useful in other areas of bioinformatics as well.

**Electronic supplementary material:**

The online version of this article (10.1186/s12859-018-2448-z) contains supplementary material, which is available to authorized users.

## Background

Protein interactions are crucial in cells for maintaining homeostasis and in regulating metabolic pathways involving thousands of chemical reactions running in parallel within an organism [1, 2]. Protein binding affinity is one of the most important aspects of protein interactions which determines protein complex stability and binding specificity and distinguishes highly specific binding partners from less specific ones [2]. Protein binding affinity is measured in terms of change in the Gibbs free energy upon binding (*∆G*). The importance of measuring binding affinity has prompted the development of various experimental techniques such as Isothermal Titration Calorimetry (ITC), Surface Plasmon Resonance (SPR), and Fluorescence Polarization (FP) which can be used to accurately measure the protein binding affinity [3–5]. However, these techniques involve laborious, time-consuming, and expensive experimental procedures and cannot be applied at a large scale. As a consequence, accurate predictive computational methods can be very useful in this domain.

Machine learning based methods are important in this area because of their ability to treat unknown factors involved in protein binding implicitly and to learn a data-driven flexible functional form [6, 7]. A number of machine learning based methods have been proposed both for predicting the absolute affinity value and to classify protein-protein complexes into low and high binding affinities using structure or sequence information [8–16]. Some of the structure-based methods give reasonable accuracy on predicting absolute binding affinities on the affinity benchmark dataset [8, 10, 11, 15]. However, these methods have limited applicability because they require 3D structures of protein complexes which are typically not available. On the other hand, state-of-the-art sequence-based methods for predicting binding affinity are not sufficiently accurate [12–14, 17, 18]. Therefore, accurate prediction of protein binding affinity using sequence information is still an unsolved problem.

**Fig. 1:**
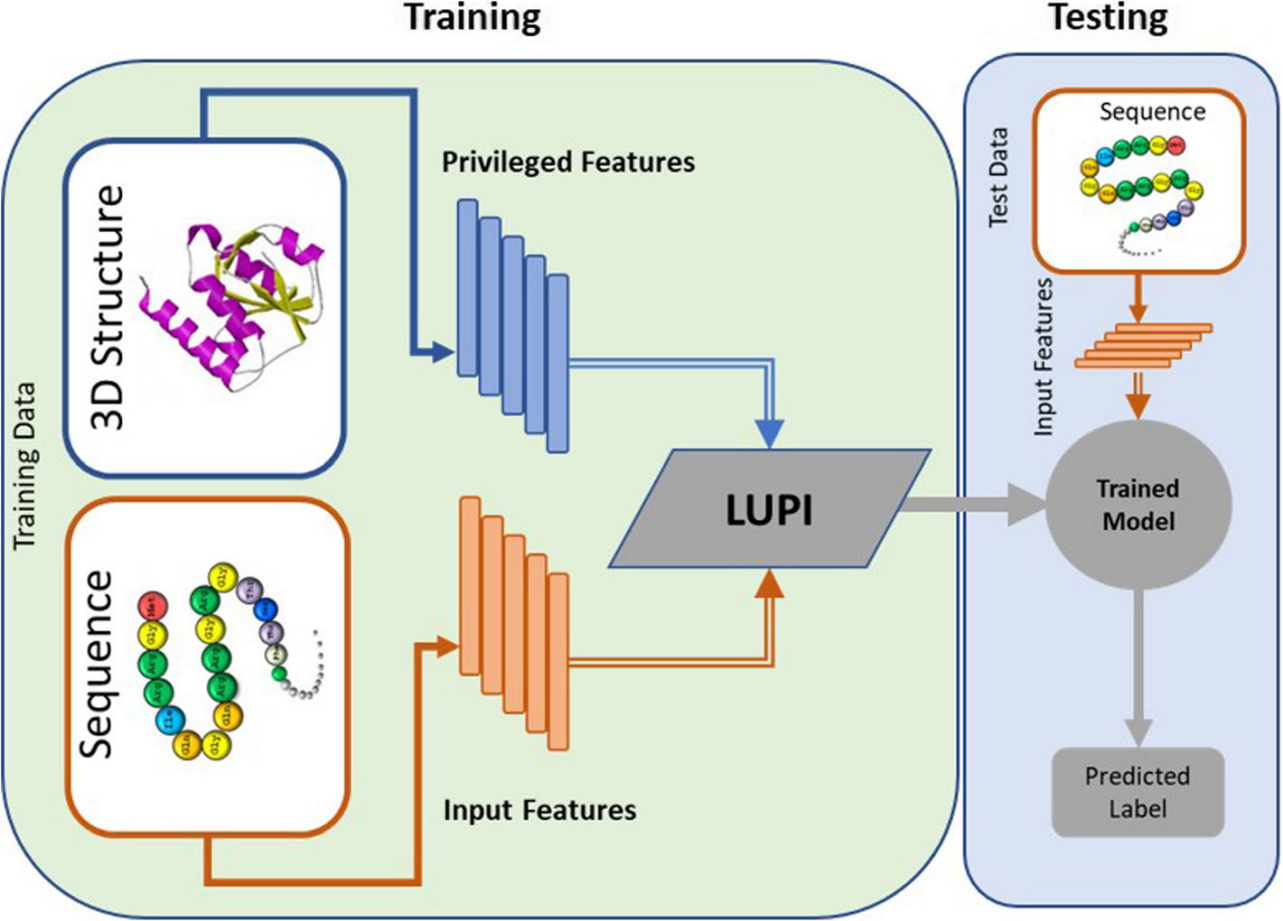
A framework to classify protein complexes based on their binding affinities with the paradigm of learning using privileged information (LUPI). Privileged information (3D structural information) is only required at training time (left panel) to help improve performance at test time using sequence information alone (right panel)

In this article, we present an implementation of the Learning Using Privileged Information (LUPI) framework for classifying protein complexes into low and high binding affinity. Our proposed method is different from previously proposed methods for the classification of protein complexes in that it uses both protein structural and sequence information during training but requires only sequence descriptors for testing (see Fig. 1). Using this method, we are able to utilize information from protein complexes with known protein 3D structures to learn a better model and still be able to predict binding affinities using sequence information alone during testing. This has led to a significant improvement in the accuracy in comparison to models which utilize only sequence information during training. We expect the LUPI framework to be very useful for other problems in bioinformatics, particularly problems that benefit from the use of protein 3D structures, such as protein function prediction and prediction of protein-protein and protein-nucleic acid interactions.

## Results

In this work, we describe a novel machine learning method to predict protein binding affinity using protein sequence and structure information. Previously, various machine learning models have been developed for this purpose using standard machine learning approaches using sequence or structure information. Typically, structure-based methods generate better predictions than sequence-based ones but are limited by the fact that structural information is not available for the vast majority of proteins. In the proposed method, we handle this constraint by following the learning using privileged information (LUPI) framework in conjunction with an SVM classifier (LUPI-SVM). In LUPI, a machine learning model is built by using additional or more informative features (called privileged space features) which are available only during training in addition to input space features that are available in both training and testing. The privileged information is expected to help the classifier converge to a better decision boundary in the input space, leading to better generalization. Applied to binding affinity prediction, LUPI-SVM uses both protein sequence and structure during training but at test time it uses only sequence-based descriptors. In what follows we present results comparing the classification performance of LUPI-SVM to several baseline classifiers to illustrate the usefulness of this approach.

### Protein binding affinity prediction with baseline learners using structure and sequence descriptors

We first compare the performance of sequence-based descriptors to structure-based ones on the task of classifying protein complexes as having low and high binding affinity. For this purpose, we have used a number of classifiers such as classical Support Vector Machines (SVMs), Random Forest (RF), and XGBoost as baseline classifiers with both sequence- and structure-based features. Results obtained with different types of structure and sequence-based features through leave one complex out (LOCO) cross-validation over the protein binding affinity benchmark dataset version 2.0 [19] which has 128 complexes, are shown in Table 1. The sequence-based features include k-mer composition and features computed using a Blosum substitution matrix to capture substitutions of physiochemically similar amino acids. For structure features, we have used Number of Interacting Residue Pairs (NIRP) to get the frequency of interacting amino acid pairs at the interface of a protein complex, Moal Descriptors which include statistical potentials, solvation and entropy terms and potentials for hydrogen bond, Dias Descriptors representing information related to binding assay pH, temperature, and methodology of determining experimental binding affinity, and Blosum-based features to capture the substitutions of physiochemically similar amino acids involved in the interface of a protein complex.

**Table 1:** Protein complex classification results obtained using classical SVM, Random Forest and XGBoost using input and privileged features with LOCO cross-validation over the affinity benchmark dataset

Features	Classical SVM			Random forest			XGBoost		
	ROC	PR	S_r_	ROC	PR	S_r_	ROC	PR	S_r_
Input space									
2-mer	**0.72**	**0.68**	**−0.40**	0.68	0.63	− 0.38	**0.72**	**0.66**	**−0.40**
Blosum (Protein)	0.70	0.63	−0.36	**0.69**	**0.62**	**−0.39**	0.69	0.63	−0.34
Privileged space									
NIRP	**0.74**	**0.71**	**−0.45**	**0.74**	**0.67**	**−0.44**	**0.72**	**0.69**	**−0.42**
Moal descriptors	0.73	0.68	−0.43	0.70	0.68	−0.37	0.71	0.68	−0.34
Dias descriptors	0.72	0.69	−0.42	0.69	0.69	−0.37	0.71	0.67	−0.34
Blosum (Interface)	0.61	0.60	−0.19	0.56	0.54	−0.11	0.66	0.59	−0.25

Bold faced values indicate best performance for each model. Blosum (Protein) refer to Blosum substitution scores averaged over the protein, while Blosum (Interface) are Blosum substitution scores averaged over the interface. Moal descriptors are taken from Moal et al. [8], and Dias descriptors are taken from Dias and Kolaczkowski [11]

*ROC* Area under the ROC curve, *PR* Area under the precision-recall curve, *S*_*r*_ Spearman correlation coefficient

The results shown in Table 1 demonstrate that structure-based features produce higher accuracy than sequence-based features for all the classifiers. For example, by using structure-based features during training and testing, we observed area under the ROC curve of 0.74 and under the precision-recall curve (PR) score of 0.71 with the number of interacting residue pairs (NIRP) as features derived from the structure of the protein complex. On the other hand, sequence-based features produce a maximum ROC score of 0.72 and PR score of 0.68 (SVM and XGBoost with 2-mer features). Moreover, we observe that most of the structural descriptors perform better than the sequence-based features. This observation suggests that structural descriptors are more informative than sequence-based features. We have also observed that performance of classical SVM is comparable to other standard state-of-the-art learners (RF and XGBoost) with different types of structure and sequence-based features. A similar trend regarding the relative performance of SVM and RF classifiers was observed by Yugandhar and Gromiha with a different dataset and evaluation protocol [13].

### Protein binding affinity prediction with LUPI-SVM using protein structural descriptors as privileged information

In this section, we present our results with the Learning Using Privileged Information (LUPI) Support Vector Machine (LUPI-SVM). LUPI-SVM uses structure-based features as privileged information which is assumed to be available only during training in conjunction with sequence-based descriptors which are used in both training and testing. Our hypothesis is that due to its modeling of structural information, LUPI-SVM will produce better accuracy for prediction of binding affinity while overcoming the limitation of predictors that require structure information during testing.

**Fig. 2:**
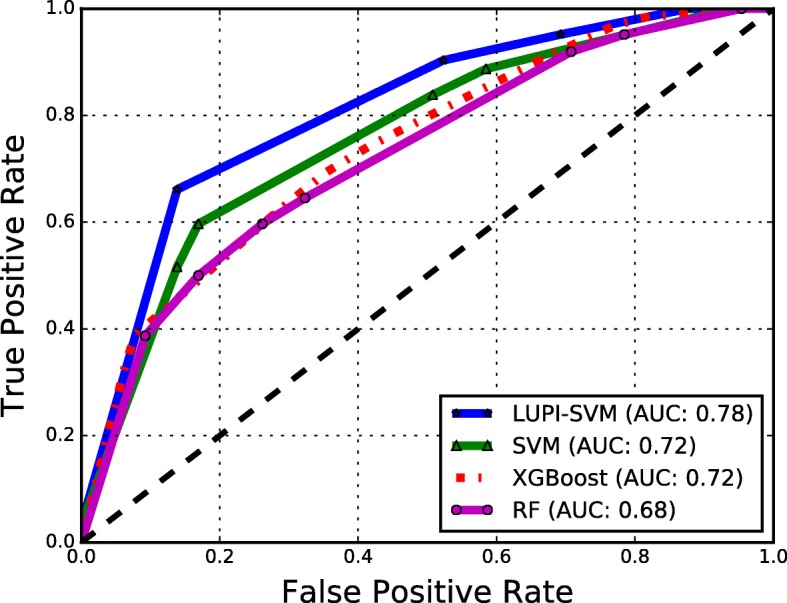
ROC curves showing a performance comparison between LUPI-SVM (with 2-mers as input-space features and Moal Descriptors as the privileged features) and the baseline classifiers (XGBoost, classical SVM (SVM), and Random Forest (RF) with 2-mer features). The average area under the ROC curve (AUC) is shown in parenthesis

The results obtained with the LUPI-SVM framework using LOCO cross-validation on the affinity benchmark dataset are shown in Table 2 and Fig. 2. In LUPI-SVM we used sequence-based features (2-mer and Blosum substitution scores averaged over the protein) as input and structure-based descriptors (NIRP, Moal Descriptors, Dias Descriptors, and Blosum substitution scores averaged over the interface) as privileged features, i.e., both sequence and structure features were used in training the classifier but only sequence-based features in testing. In Table 2 we have also show results of classical SVM using sequence-based features for an easy comparison with LUPI-SVM. An area under the ROC curve (ROC) score of 0.78 and under the precision-recall curve (PR) score of 0.73 were obtained using Moal Descriptors as privileged information and 2-mer features as input-space features; this is a large improvement over the best baseline SVM performance of 0.72 and 0.68 for area under the ROC curve and PR curve, respectively. In all cases, the use of privileged information led to improved performance, even when using the Blosum substitution scores averaged over the interface, that had lower performance than sequence-based features. Surprisingly, the performance of LUPI-SVM was also slightly higher than an SVM which used privileged structure information for both training and testing. This suggests that LUPI-SVM can make effective use of both sources of information and provide performance that is better than both sources by themselves. Moreover, it is worth noting that the best features as privileged features are not the ones that give the best performance on the classification task, and that Moal descriptors consistently provided the best performance as privileged features.

**Table 2:** Protein complex classification results obtained through classical SVM and LUPI across different features using LOCO cross-validation over the affinity benchmark dataset

	Input features					
	2-mer			Blosum (Protein)		
	ROC	PR	*S*_*r*_	ROC	PR	*S*_*r*_
	Classical SVM					
	**0.72**	**0.68**	**−0.40**	0.70	0.63	−0.36
Privileged features	LUPI-SVM					
NIRP	0.76	0.71	−0.47	0.74	0.70	−0.42
Moal descriptors	**0.78**	**0.73**	**−0.48**	**0.75**	**0.73**	**−0.43**
Dias descriptors	0.74	0.70	−0.45	0.73	0.69	−0.40
Blosum (Interface)	0.73	0.69	−0.41	0.73	0.69	−0.42

Bold faced values indicate best performance for each model

*ROC* Area under the ROC curve, *PR* Area under the precision-recall curve, *S*_*r*_ Spearman correlation coefficient

In order to test the performance of the proposed scheme in predicting binding affinity of different types of protein complexes, we have also computed the performance of LUPI-SVM across three major classes of complexes in the dataset. We observed area under the precision-recall curve (PR) score of 0.68, 0.58 and 0.82 and area under the ROC curve (ROC) score of 0.82, 0.67 and 0.71 for enzyme containing (E), antibody/antigen (A), and other complexes (O), respectively using Moal Descriptors as privileged information and 2-mer features as input-space features. These results also show a significant improvement in comparison to baseline SVM performance in terms of PR score of 0.62, 0.42 and 0.80 and ROC score of 0.72, 0.57 and 0.69 for enzyme containing (E), antibody/antigen (A), and other complexes (O), respectively.

We have also divided complexes into rigid, medium and difficult classes on the basis of conformational change upon complex formation. We have observed an improved performance of LUPI-SVM for rigid and medium complexes with area under the precision-recall curve (PR) score of 0.74, 0.84 and area under the ROC curve (ROC) score of 0.82 and 0.92, in comparison to the baseline SVM with PR score of 0.69, 0.78 and ROC score of 0.73 and 0.84, respectively. For difficult complexes, both LUPI-SVM and the baseline SVM exhibited the same performance with PR score of 0.85 and ROC score of 0.74.

### Performance comparison of LUPI-SVM and models build using classical machine learning setting on the independent validation dataset

In addition to performance comparison using LOCO cross-validation, we have also used an additional validation set to compare different machine learning models with the proposed LUPI-SVM approach. This dataset contains 12 positive examples (high affinity) and 27 negative examples (low affinity) and has no overlap with the affinity benchmark dataset. In this case, we trained the baseline and LUPI-SVM models on the affinity benchmark dataset and tested on the validation dataset. The results are shown in Table 3. Using a classical SVM, we obtained a maximum area under the ROC curve (ROC) score of 0.63 and precision-recall curve (PR) score of 0.38 using 2-mer features, whereas by using LUPI-SVM trained using 2-mer as input features and Moal descriptors as privileged information, we obtained a much higher ROC score of 0.71 and PR score of 0.46. This shows an improved performance of LUPI-SVM over the classical SVM.

**Table 3:** Comparison of classical SVM and LUPI-SVM on the external independent validation dataset with training on affinity benchmark dataset

	Input features					
	2-mer			Blosum (Protein)		
	ROC	PR	*S*_*r*_	ROC	PR	*S*_*r*_
	Classical SVM					
	0.63	0.38	− 0.28	0.61	0.39	−0.19
Privileged features	LUPI-SVM					
NIRP	0.66	0.42	−0.30	0.64	0.40	−0.28
Moal descriptors	**0.71**	**0.46**	**−0.39**	**0.69**	**0.48**	**−0.30**
Dias descriptors	0.65	0.41	−0.29	0.64	0.44	−0.20
Blosum (Interface)	0.64	0.40	−0.26	0.64	0.46	−0.22

Bold faced values indicate best performance for each model

*ROC* Area under the ROC curve, *PR* Area under the precision-recall curve, *S*_*r*_ Spearman correlation coefficient

We have also used this validation set to compare LUPI-SVM against the existing state-of-the-art method for protein affinity prediction called PPA-Pred2 [12] using its webserver (accessed: March 18, 2018). For this comparison, we obtained predictions for the complexes in our validation dataset from the PPA-Pred2 webserver and computed the ROC score based on the predicted binding affinity values. We obtained a ROC score of 0.63 compared to 0.71 using the proposed LUPI-SVM method. The low performance of PPA-Pred2 on this validation dataset has already been reported independently by Moal et al., [17, 18] as well. These results provide further support for the advantage of using protein structural information as privileged information in the LUPI framework.

### Feature analysis for binding affinity prediction

**Fig. 3:**
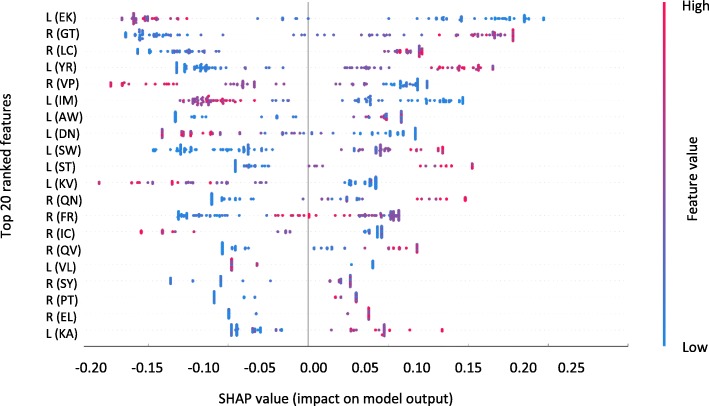
Feature analysis using SHAP. The impact of 2-mer features on model output is shown using SHAP values. The plot shows the top 20 2-mers for the Ligand (L) or Receptor (R) by the sum of their SHAP values over all samples. Feature value is shown in color (Red: High; Blue: Low) reveals for example that a high value of L (EK) (Counts of ‘EK’ in a protein sequence designated as ligand) contributes more for predicting low binding affinity complexes

To discover the features that contribute to predicting binding affinity, we used the SHapley Additive exPlanations (SHAP) tool [20]. SHAP values reveal the importance of a feature in predicting binding affinity: for example, a high SHAP value of the count of the amino acid pair EK in the ligand proteins (denoted by L (EK) in Fig. 3) indicates that the existence of EK contributes more for predicting low binding affinity complexes. Similarly, R (GT) (Counts of ‘GT’ mer in a protein sequence designated as receptor) contributes more for predicting high binding affinity complexes (see Fig. 3).

Different types of amino acids are involved in these top 20 2-mers such as lysine (K), Glutamic Acid (E), Arginine (R), Aspartic Acid (D), Leucine (L), Tryptophan (W), Tyrosine (Y) and Serine (S). In the top 20 2-mers, Tryptophan (W), Tyrosine (Y), Serine (S), Thyronine (T) and Arginine (R) are involved in those 2-mers which contribute more in predicting high binding affinity complexes. These amino acids have already been highlighted as hot spots in protein interactions in previous studies [21, 22].

### Learned models using LUPI and classical SVM

**Fig. 4:**
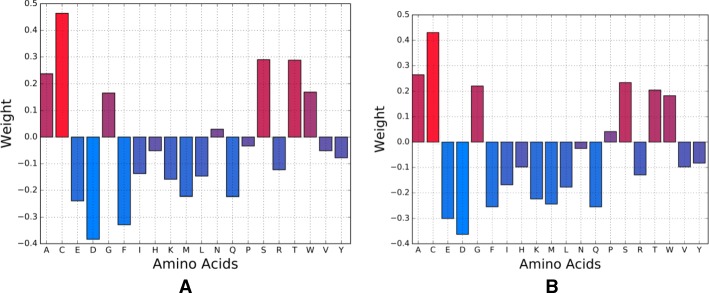
Weight vectors of the trained classifiers for the ligand Blosum features. **a** SVM with LUPI framework using Blosum substitution features computed over each protein as input and Moal Descriptors as privileged features; **b** Classical SVM using Blosum features

We have used weight vectors of the best-trained models using both LUPI-SVM and classical SVM to get insight into the role of privileged information in training. Figure 4 shows the weight vector of the trained classifier for the ligand Blosum features using both LUPI-SVM and classical SVM. Overall, both models show similar contributions of each residue, and the role of privileged information in LUPI-SVM appears to be in fine-tuning the weights for improved accuracy.

## Discussion

Computational protein binding affinity prediction techniques are important for determining the binding specificity of proteins and their interactions due to the difficulty of obtaining this information experimentally. Among these computational methods, a number of machine learning methods have been proposed which use both protein sequence and structures. All the available machine learning methods operate in the setting where information used during training should be available in in the same way during testing. This requirement limits the applicability of methods trained using protein 3D structure, as most proteins do not have solved 3D structures. We have also observed that training models using sequence information only and ignoring structural information results in a loss of accuracy. It turns out that it is possible to have the best of both worlds and obtain even better performance than either source of data on its own, while still only requiring sequence information during testing using the proposed LUPI-SVM method. Improved performance of LUPI-SVM over the baseline classifiers and existing state-of-the-art method (PPA-Pred2) [12] suggests that the proposed method can effectively use both sources of information.

## Conclusions and future work

We presented a novel machine learning method for protein affinity prediction that uses both protein structure and sequence information during training but needs only sequence information for testing. To the best of our knowledge, this is first attempt to combine protein structure and sequence information in this way to predict binding affinity. A comparison of the proposed LUPI-SVM framework with different baseline learners and a state-of-the-art binding affinity predictor shows that our proposed method not only performs better in cross-validation but also on an additional validation dataset. However, there is still a large room of improvement in protein affinity prediction. As already suggested in a recent study by Dias and Kolaczkowski, to achieve better performance in this domain, we need either a significant increase in the amount of quality affinity data or methods of leveraging data from similar problems [11].

A number of other problems in bioinformatics in which the existence of structure data is a bottleneck, can be addressed by combining sequence and 3D structural information using the framework of learning using privileged information. These include protein function prediction [23], protein-DNA, protein-RNA and protein-protein interaction prediction [24–27] as well. Finally, we expect that the freely available Python implementation of the LUPI-SVM framework will be helpful for applications in other problem domains.

## Methods

### Dataset and preprocessing

In this study we have used the protein binding affinity benchmark dataset 2.0 [19]. This dataset is a subset of docking benchmark version 4.0 (DBD-4.0) and contains 144 non-redundant protein complexes with solved bound and unbound 3D structures of the ligand and receptor proteins at an average resolution of 1.2 Å (min: 0.17 Å; max: 4.9 Å) [19, 28]. Protein complexes in this dataset have known binding affinities in terms of binding free energy and disassociation constant and have been divided into three major groups: (A) antibody/antigen, (E) enzyme containing, and (O) other complexes. The binding free energy ranges from − 18.58 to − 4.29. One protein complex (CID: 1NVU) in this dataset has two entries due to allostery [19, 29]. We considered only one of them (1NVU_Q: S), with an affinity value of − 7.43, due to lack of availability of structural information of interacting chains of the second entry. Following the same data curation and preprocessing technique used by Moal et al., and Yugandhar and Gromiha, we have selected 128 complexes (for detail see in the Additional file 1: Table S1) from this dataset after removing those complexes which: have a protein with length less than 50 amino acids, are not heterodimeric and have difficulty of deriving a full structural feature set [8, 12]. This allows us to use descriptors from Moal et al. and Dias et al., [8, 11]. We have divided this dataset into two parts: complexes with low binding affinity (65 complexes) and complexes with high binding affinity (63 complexes) using a threshold − 10.86 which is median value of binding affinity in our data set and has been used in other studies as well [13].

We have also used an external validation dataset of 39 protein-protein complexes with known binding free energy to perform a stringent performance comparison of different methods and machine learning models. This dataset is derived from Chen et al. by removing complexes having more than two chains and involving chains of size less than 50 residues [30]. This dataset has been used for validation in a related study [17]. We have also used this dataset to compare the performance of our proposed method with PPA-Pred2 [12] by using the predicted binding affinity values obtained from its webserver, which is available at https://www.iitm.ac.in/bioinfo/PPA_Pred/, accessed on 18-03-2018.

### Classifiers for prediction of binding affinity

We propose a machine learning approach for the classification of protein-protein complexes based on their binding affinities using both structure and sequence information. As discussed earlier, the novelty of the proposed approach is that it uses both sequence and structure of protein during training time but requires only sequence information during testing (see Fig. 1). The proposed scheme is based on the paradigm of learning using privileged information (LUPI) [31].

In this work, we formulate binding affinity as a classification problem: classifying protein complexes as having low or high binding affinity. Thus, our dataset consists of examples of the form (***c***_***i***_, ***y***_***i***_) where ***c***_***i***_ is a protein complex and ***y***_***i***_ ∈ {+1, −1} is its associated label indicating whether ***c***_***i***_ has binding free energy less than −10.86 (+1) or not (−1). The threshold −10.86 is the median value of binding affinity in our data set and has been used in other studies as well [13]. This results in 63 high binding affinity complexes (with label +1) and 65 low binding affinity complexes (with label −1). For a given protein complex ***c***_***i***_, we extract sequence and structure-based features from it which are denoted by ***x***_***i***_ and ${\boldsymbol{x}}_i^{\prime }$, respectively. Our objective is to learn a function that classifies a given protein complex into high or low affinity using sequence information alone.

#### Baseline classifiers

As a baseline, we have used three different classifiers: classical Support Vector Machine (SVM), Random Forest (RF) and Gradient Boosting Machine (XGBoost) [32–35].

#### Classical Support Vector Machine (SVM)

We used SVM to classify a protein complex into high or low binding affinity by learning a function *f*(***x***) = 〈***w***, ***x***〉 with ***w*** as parameters to be learned from the training data {(***x***_***i***_, *y*_*i*_ )| *i* = 1, 2, …, *N*}. Optimal value of the ***w*** is obtained in SVM by solving the following optimization problem [32].

1 $$ {\min}_{\boldsymbol{w},\xi}\frac{1}{2}\lambda \parallel \boldsymbol{w}{\parallel}^2+\sum \limits_{i=1}^N{\upxi}_i $$

Subject to:

$$ {y}_i\left\langle \boldsymbol{w},{\boldsymbol{x}}_i\right\rangle \ge 1-{\xi}_i,{\xi}_i\ge 0,\forall i=1,\dots, N. $$

The objective function in Eq. (1) maximizes the margin while minimizing margin violations (or slacks ***ξ***) [32]. The hyperparameter $\lambda =\frac{1}{C}$ controls the tradeoff between margin maximization and margin violation. We used both linear and radial basis function (RBF) kernels and coarsely optimized the values of *λ* and *γ* using grid search with scikit-learn (version:0.18) [36].

#### Random Forest

Random Forest (RF) is an ensemble learning method that operates by constructing multiple decision trees on random subsamples of input features and examples and classifies an example using a majority vote [33]. Random Forests have been used in many related studies [13, 14]. Hyperparameter selection was performed with respect to the number of trees and the minimum number of examples required for a split and used the implementation available in scikit-learn (version:0.18) [36].

#### Gradient Boosting (XGBoost)

Gradient boosting is also an ensemble learning method; it combines weak learners into a strong learner in an iterative fashion [34, 35]. We have performed model selection for XGBoost in terms of the number of boosting iterations, booster, subsample ratio, learning rate, and maximum depth using a grid search and xgboost 0.7 [35].

#### LUPI-SVM

The LUPI-SVM framework was recently proposed by Vapnik and Izmailov [31]. Like the standard SVM, this model also learns a linear discriminant function *f*(***x***_***i***_) = 〈***w***, ***x***_***i***_〉 in the input space. However, in LUPI, instead of slack variables as in a standard SVM, we have a slack function ${\xi}_{\boldsymbol{i}}=\left\langle {\boldsymbol{w}}^{\prime },{\boldsymbol{x}}_{\boldsymbol{i}}^{\prime}\right\rangle$ based on the privileged features. This controls the decision boundary in the input space using information from privileged features. In LUPI, we learn ***w*** by using training data of the form $\left\{\left({\boldsymbol{x}}_i,{\boldsymbol{x}}_i^{\prime },{y}_i\ \right)|i=1,\dots, N\right\}$ where, **x**_***i***_ and **x**′_***i***_ are feature vectors for protein complex *c*_*i*_ belonging to the input and privileged feature spaces, respectively, and *y*_*i*_ ∈ {+1, −1} is the associated label. The mathematical formulation of the LUPI-SVM can be written as:

2 $$ {\min}_{\boldsymbol{w},{\boldsymbol{w}}^{\prime },{\xi}^{\prime }}\frac{1}{2}\left[\lambda \parallel \boldsymbol{w}{\parallel}^2+{\lambda}^{\prime}\parallel {\boldsymbol{w}}^{\prime }{\parallel}^2\right]+{\lambda}^{\prime \prime}\sum \limits_{i=1}^N\left[{y}_i\left\langle {\boldsymbol{w}}^{\prime },{\boldsymbol{x}}_i^{\prime}\right\rangle +{\xi}_i^{\prime}\right]+\sum \limits_{i=1}^N{\xi}_i^{\prime } $$

Subject to:

$$ {y}_i\left\langle \boldsymbol{w},{\boldsymbol{x}}_i\right\rangle \ge 1-\left[{y}_i\left\langle {\boldsymbol{w}}^{\prime },{\boldsymbol{x}}_i^{\prime}\right\rangle +{\xi}_i^{\prime}\right], $$

$$ {y}_i\left\langle {\boldsymbol{w}}^{\prime },{\boldsymbol{x}}_i^{\prime}\right\rangle +{\xi}_i^{\prime}\ge 0, $$

$$ {\xi}_i^{\prime}\ge 0,\forall i=1,\dots, N $$

where, *λ*, *λ*^′^, and *λ*^′′^ are hyper-parameters which control the trade-off between margin maximization and margin violations. Slack variables in the privileged space *ξ*^′^ enforce the constraint that input-space slack values are non-negative.

In order solve this optimization problem, we have developed a stochastic sub-gradient optimization (SSGO) algorithm inspired by the Pegasos solver for binary SVMs [37]. To do so, we write the constrained optimization problem in Eq. (2) as an unconstrained one as follows:

3 $$ {\min}_{\boldsymbol{w},{\boldsymbol{w}}^{\prime }}\frac{1}{2}\left[\lambda \parallel \boldsymbol{w}{\parallel}^2+{\lambda}^{\prime}\parallel {\boldsymbol{w}}^{\prime }{\parallel}^2\right]+{\lambda}^{\prime \prime}\sum \limits_{i=1}^N{y}_i\left\langle {\boldsymbol{w}}^{\prime },{\boldsymbol{x}}_i^{\prime}\right\rangle +\sum \limits_{i=1}^Nl\left({y}_i,f\left({\boldsymbol{x}}_i;{\boldsymbol{x}}_i^{\prime },\boldsymbol{w},{\boldsymbol{w}}^{\prime}\right)\right) $$

with the loss function:

$$ l\left({y}_i,f\left({\boldsymbol{x}}_i;{\boldsymbol{x}}_i^{\prime },\boldsymbol{w},{\boldsymbol{w}}^{\prime}\right)\right)=\max \left\{0,-{y}_i\left\langle {\boldsymbol{w}}^{\prime },{\boldsymbol{x}}_i^{\prime}\right\rangle, 1-{y}_i\left\langle \boldsymbol{w},{\boldsymbol{x}}_i\right\rangle -{y}_i\left\langle {\boldsymbol{w}}^{\prime },{\boldsymbol{x}}_i^{\prime}\right\rangle \right\}. $$

The stochastic sub-gradient solver for this problem operates iteratively by choosing a protein complex randomly in each iteration and estimating the sub-gradient of the objective function given in Eq. (3) based only on the chosen complex. The sub-gradient at iteration *t* can be written as:

$$ {\mathbf{\nabla}}_t=\Big\{{\displaystyle \begin{array}{cc}\lambda {\boldsymbol{w}}^T-{y}_t{\boldsymbol{x}}_t& if{y}_t\left\langle \boldsymbol{w},{\boldsymbol{x}}_t\right\rangle +{y}_t\left\langle {\boldsymbol{w}}^{\prime },{\boldsymbol{x}}_t^{\prime}\right\rangle >1 and1-{y}_t\left\langle \boldsymbol{w},{\boldsymbol{x}}_t\right\rangle >0\\ {}\lambda {\boldsymbol{w}}^T& otherwise\end{array}}\operatorname{} $$

$$ {\mathbf{\nabla}}_t^{\prime }=\Big\{{\displaystyle \begin{array}{cc}{\lambda}^{\prime }{\boldsymbol{w}}^{\prime^T}+{\lambda}^{\prime \prime }{y}_t{\boldsymbol{x}}_t^{\prime }-{y}_t{\boldsymbol{x}}_t^{\prime }& if-{y}_t\left\langle {\boldsymbol{w}}^{\prime },{\boldsymbol{x}}_t^{\prime}\right\rangle >0 or{y}_t\left\langle \boldsymbol{w},{\boldsymbol{x}}_t\right\rangle +{y}_t\left\langle {\boldsymbol{w}}^{\prime },{\boldsymbol{x}}_t^{\prime}\right\rangle >1\\ {}{\lambda}^{\prime }{\boldsymbol{w}}^{\prime^T}+{\lambda}^{\prime \prime }{y}_t{\boldsymbol{x}}_t^{\prime }& otherwise\end{array}}\operatorname{} $$

**Fig. 5:**
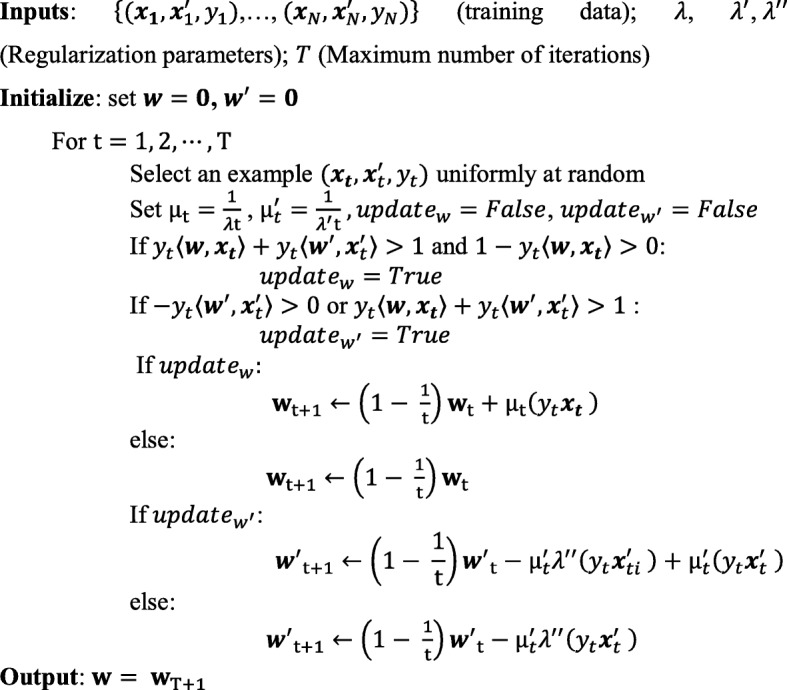
Training algorithm for LUPI-SVM with stochastic sub-gradient optimization

The weight vectors are updated in a direction opposite to the direction of the sub-gradient by the following equations

$$ {\boldsymbol{w}}_{t+1}\leftarrow {\boldsymbol{w}}_t-{\mu}_t{\mathbf{\nabla}}_t $$

$$ {\boldsymbol{w}}_{t+1}^{\prime}\leftarrow {\boldsymbol{w}}_t^{\prime }-{\mu}_t^{\prime }{\mathbf{\nabla}}_t^{\prime } $$

using a step size of ${\mu}_t=\frac{1}{t\lambda}$ and ${\mu}_t^{\prime }=\frac{1}{{t\lambda}^{\prime }}$ . The complete optimization algorithm is given in Fig. 5. Our python-based implementation of learning using privileged information algorithm is available online at: https://github.com/wajidarshad/LUPI-SVM.

### Feature representations

In this work we have used both structure- and sequence-based feature representations. The sequence-based features are used as input space features whereas structural features are used as privileged space features, i.e., it is assumed that structural features are available only for training. All feature representations are standardized to zero mean and unit variance across all complexes. The details of feature representation are as follows.

#### Sequence-based features

In order to model the sequence-based attributes of a protein complex containing ligand and receptor chains, we first obtain sequence-based features of all chains in the ligand and receptor separately. The features of all chains in the ligand (or receptor) are then averaged across chains to get a single feature vector for the ligand (or receptor). The feature vector representations of ligand and receptor are then concatenated to produce a feature vector for the protein complex as performed elsewhere [38]. We give details of the individual chain level sequence-based feature descriptors used in this study below.

#### k-mer composition (k-mer)

k-mer composition i.e. the counts of the occurrences of k-mers in a protein sequence, is a widely used descriptor of a protein sequence [39]. We used this feature representation to capture the composition of a protein sequence. For k-mers of size 2 (2-mer) this yields a 400-dimensional feature representation of each protein chain.

#### BLOSUM-62 features: Blosum (Protein)

In order to represent amino acid composition and at the same time capture substitutions of physiochemically similar amino acids in a protein sequence, a protein sequence is converted into a 20-dimensional vector by averaging the columns from a BLOSUM substitution matrix corresponding to each amino acid in a given sequence. We used a BLOSUM-62 substitution matrix to extract this feature representation [40]. This feature representation has already been used successfully in several related studies [41–44].

#### Structure-based features (privileged feature space)

Proteins interact and perform their function through their 3D structure. Therefore, structural properties of a protein complex play a vital role in defining the binding affinity of a protein complex. In order to extract structural properties of a protein complex, we used different complex level feature representations. We have used these features both as a baseline and for LUPI as privileged information. Different type of structural feature representations of each complex in our dataset used in this study are described below.

#### Number of interacting residue pairs (NIRP)

**Fig. 6:**
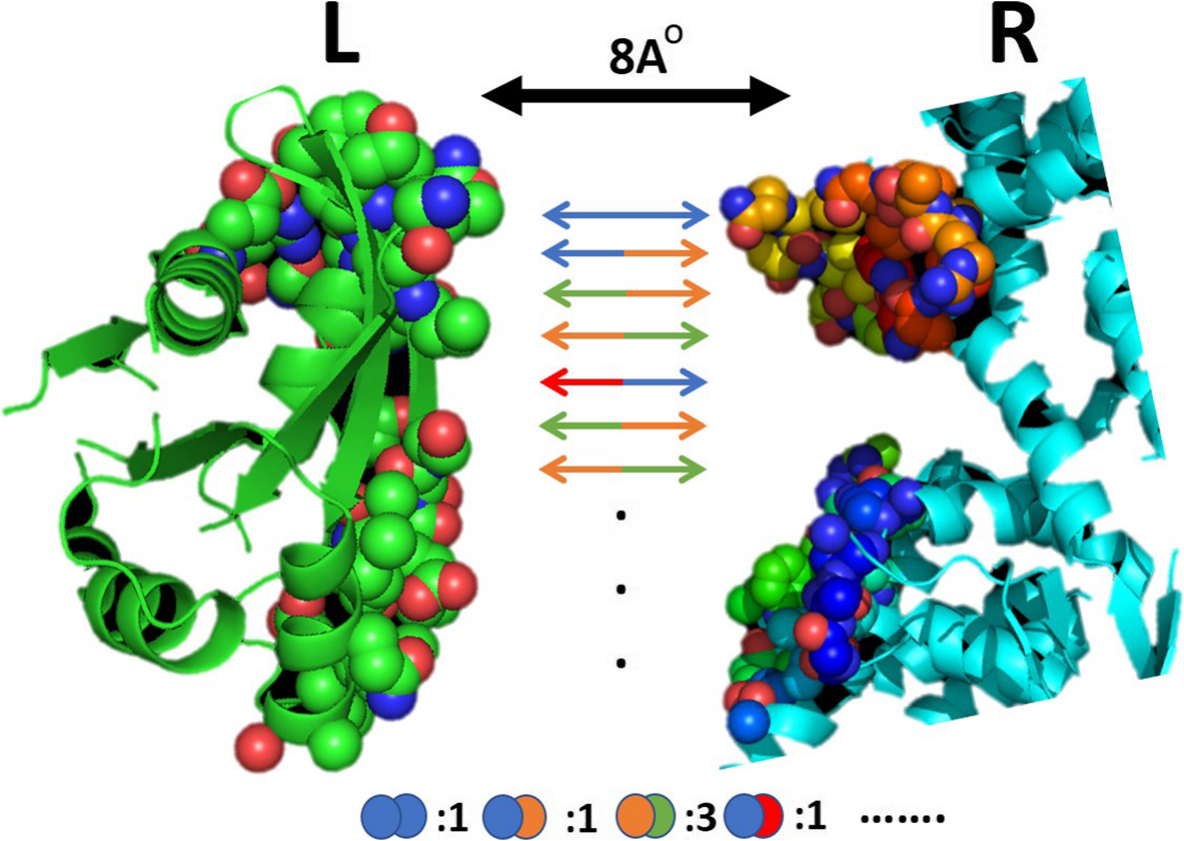
Number of interacting residue pairs (NIRP) in the interface of a protein complex. The frequency of non-repeating pairs (considering A: B and B: A the same) was computed from the bound 3D structures of ligand (L) and receptor (R) of a protein complex. Residues (shown as spheres) at a distance cutoff of 8 Å are considered the interface of the complex. The bottom panel of the figure shows the form of the feature vector extracted using this scheme

Interactions in a protein-protein complex are normally stabilized by the non-covalent interaction between residues occurring at the interface of ligand and receptor [45]. The amino acids involved in these non-covalent interactions at the interface of a protein complex determine the binding mode and binding energy of a protein complex [1]. For this reason, we used the frequency of interacting amino acids pairs at the interface of a protein complex as shown in Fig. 6. Through this method, we extracted a 211-dimensional feature representation from the bound structures of ligand and receptor of a protein complex using a distance cutoff of 8 Å.

#### Moal descriptors

These descriptors were obtained from a study on protein-protein binding affinity prediction by Moal et al., [8]. This 200-dimensional feature representation of a complex describes the interface and conformational changes upon binding. These features include statistical potentials (residue and atomic pair potentials, four-body potentials), solvation and entropy terms (atomic contact energies, continuum electrostatics models, hydrophobic burial, terms for translational, rotational, vibrational, side chain and disorder to order transition entropies), unbound-bound descriptors (change in internal energy) and other potential terms like energy terms associated with electrostatics, London dispersion and exchange repulsion forces, as well as potentials for hydrogen bond [8]. By using these descriptors, a correlation score of 0.55 has been reported between the experimental and predicted binding affinities of the complexes in the affinity benchmark dataset [8].

#### Dias descriptors

We obtained these descriptors from a study on protein-protein binding affinity prediction by Dias and Kolaczkowski [11]. These descriptors include information related to binding assay pH, temperature, and methodology of determining experimental binding affinity value of each complex in the benchmark dataset [11]. We have converted the string values of experimental methods into a feature vector using binary one-hot encoding [46]. These descriptors give a 27-dimensional feature representation for each complex in our dataset. A Pearson correlation of 0.68 between the experimental and predicted binding affinities has been reported using these descriptors [11].

#### Average BLOSUM-62 features: Blosum (Interface)

As discussed earlier, we extracted Blosum features to model substitution of physiochemically similar amino acids in a protein sequence. We have also extracted this feature representation for amino acids involved in the interface of a protein complex with a distance cutoff of 8 Å.

### Model validation, selection and performance assessment

We used Leave One Complex Out (LOCO) cross-validation to evaluate our classification models over the non-redundant binding affinity benchmark dataset [8]. This scheme of cross-validation allows us to include more training data by developing the model with (*N* – 1) complexes and testing on the left out complex. This process is repeated for all the complexes in the dataset to get a single value of an accuracy metric. We have used area under the ROC curve (ROC), area under the precision-recall curve (PR) and the Spearman correlation coefficient (*S*_*r*_) as accuracy metrics for model evaluation and performance assessment [44, 47–49]. Average values of all the metrics obtained by shuffling the data across 3 runs of LOCO cross-validation have been reported in the results and discussion section.

In order to get the optimal values of the hyperparameters for all the baseline classifiers and LUPI-SVM, we used grid search with an area under the ROC curve as the metric for selection with nested 5-fold cross-validation. For the standard SVM, the range of values for *λ* and *γ* was [10^−3^, 10^3^ ] and [10^−3^, 10^1^ ], respectively. Similarly, for LUPI-SVM, we used values for *λ*, *λ*^′^ and *λ*^′′^ in the range [10^−5^, 10^3^ ]. We used the best hyperparameters selected through grid search for the training and testing of the final model.

## Additional file

Additional file 1: **Table S1.** Detail of 128 protein complexes with known binding affinity values used as training dataset. (DOCX 34 kb)

## Abbreviations

LOCO: Leave one complex out
LUPI: Learning using privileged information
PR: Area under the precision-recall curve
RF: Random Forests
ROC: Area under the ROC curve
