# Supplementary material for: Learning protein binding affinity using privileged information
Source: BMC Bioinformatics. 2018 Nov 15;19:425. doi: 10.1186/s12859-018-2448-z (PMC6238365; doi:10.1186/s12859-018-2448-z)
Supplement: Supplementary file 1 — Table S1. Detail of 128 protein complexes with known binding affinity values used as training dataset. (DOCX 34 kb) [file 12859_2018_2448_MOESM1_ESM.docx]

**Learning Protein Binding Affinity using Privileged Information**

**Wajid Arshad Abbasi**^1,2,3^**, Amina Asif**^1^**, Asa Ben-Hur**^3, *^ **and Fayyaz ul Amir Afsar Minhas**^1,*^

^1^Biomedical Informatics Research Laboratory (BIRL), Department of Computer and Information Sciences (DCIS), Pakistan Institute of Engineering and Applied Sciences (PIEAS), Nilore, ISL 45650, Pakistan.

^2^Information Technology Center (ITC), University of Azad Jammu & Kashmir, Muzaffarabad, 13100, Azad Kashmir, Pakistan.

^3^Department of Computer Science, Colorado State University (CSU), Fort Collins, CO 80523, USA.

***Corresponding author’s email:** [asa@cs.colostate.edu](mailto:asa@cs.colostate.edu) or [afsar@pieas.edu.pk](mailto:afsar@pieas.edu.pk)

**Additional file 1**

—————————— ◆ ——————————

**Contents**

1. **Table S1.** Detail of 128 protein complexes with known binding affinity values used as training dataset ---------------------------------------------------------------------------02

| **Table S1. Detail of 128 protein complexes with known binding affinity values used as training dataset** | | | | | | | |
| --- | --- | --- | --- | --- | --- | --- | --- |
| **S.#** | **Complex ID** | **Class** | **Affinity value** | **S.#** | **Complex ID** | **Class** | **Affinity value** |
| 1 | 1JPS | A | -13.64 | 65 | 3SGB | E | -14.51 |
| 2 | 1KTZ | O | -8.92 | 66 | 2O3B | E | -15.68 |
| 3 | 2SIC | E | -13.84 | 67 | 2B4J | O | -10.86 |
| 4 | 2HRK | O | -10.98 | 68 | 2TGP | O | -7.54 |
| 5 | 1XD3 | O | -8.9 | 69 | 1FLE | E | -12.28 |
| 6 | 1EAW | E | -14.06 | 70 | 2AJF | O | -10.63 |
| 7 | 1VFB | A | -11.46 | 71 | 1KAC | O | -10.68 |
| 8 | 1E4K | O | -7.87 | 72 | 1JTG | E | -12.82 |
| 9 | 1I4D | O | -7.46 | 73 | 1QA9 | O | -7.16 |
| 10 | 1MQ8 | O | -7.53 | 74 | 1AHW | A | -11.55 |
| 11 | 1OC0 | E | -12.28 | 75 | 1T6B | O | -13.1 |
| 12 | 1H1V | O | -10.2 | 76 | 1HCF | O | -13.08 |
| 13 | 2PCC | E | -7.91 | 77 | 2OZA | O | -11.73 |
| 14 | 2PCB | O | -6.82 | 78 | 2J0T | E | -13.34 |
| 15 | 1H9D | O | -9.18 | 79 | 1EMV | E | -18.58 |
| 16 | 2HLE | O | -10.09 | 80 | 1OPH | E | -11.32 |
| 17 | 1FQJ | O | -9.79 | 81 | 2AQ3 | O | -6.71 |
| 18 | 2OOB | E | -5.66 | 82 | 1XQS | O | -7.08 |
| 19 | 1RV6 | O | -13.86 | 83 | 1ZLI | E | -12.04 |
| 20 | 2OOR | E | -10.65 | 84 | 2PTC | E | -18.04 |
| 21 | 1KLU | O | -7.28 | 85 | 1Z0K | O | -6.98 |
| 22 | 1WQ1 | O | -6.62 | 86 | 1R0R | E | -14.17 |
| 23 | 1GXD | E | -11.3 | 87 | 1F6M | E | -7.6 |
| 24 | 2NYZ | O | -12.69 | 88 | 1WEJ | A | -12.48 |
| 25 | 1NW9 | E | -11.19 | 89 | 1ACB | E | -13.05 |
| 26 | 1GRN | O | -9.03 | 90 | 3BZD | O | -9.57 |
| 27 | 1CBW | O | -10.75 | 91 | 1KXP | O | -12.34 |
| 28 | 1ATN | O | -12.07 | 92 | 1KXQ | A | -11.54 |
| 29 | 1WDW | E | -12.72 | 93 | 1GCQ | O | -6.51 |
| 30 | 1LFD | O | -7.79 | 94 | 2B42 | E | -12.11 |
| 31 | 2OUL | E | -11.96 | 95 | 2HQS | O | -10.15 |
| 32 | 1IJK | E | -10.42 | 96 | 1AKJ | O | -5.32 |
| 33 | 1JMO | E | -9.47 | 97 | 2JEL | A | -11.59 |
| 34 | 1PXV | E | -12.97 | 98 | 1KKL | E | -10.02 |
| 35 | 1EWY | E | -7.43 | 99 | 1FC2 | O | -10.43 |
| 36 | 1RLB | O | -8.18 | 100 | 1S1Q | O | -4.29 |
| 37 | 1US7 | E | -8.09 | 101 | 2MTA | E | -7.42 |
| 38 | 1DQJ | A | -11.67 | 102 | 2VIS | O | -7.36 |
| 39 | 1YVB | E | -11.17 | 103 | 2VIR | A | -12.28 |
| 40 | 1FFW | O | -8.09 | 104 | 1IB1 | O | -9.76 |
| 41 | 2BTF | O | -7.69 | 105 | 1E6J | A | -10.28 |
| 42 | 2I25 | A | -12.28 | 106 | 1JIW | E | -15.55 |
| 43 | 1I2M | O | -15.83 | 107 | 3CPH | O | -8.84 |
| 44 | 1BUH | E | -9.7 | 108 | 1EZU | E | -13.77 |
| 45 | 3BP8 | O | -11.44 | 109 | 1E6E | E | -8.28 |
| 46 | 1EFN | O | -10.12 | 110 | 2C0L | O | -9.82 |
| 47 | 1DFJ | E | -18.05 | 111 | 1GPW | O | -11.32 |
| 48 | 2UUY | E | -11.26 | 112 | 1B6C | O | -8.94 |
| 49 | 2VDB | O | -13.4 | 113 | 1ZHI | O | -9.08 |
| 50 | 1PVH | O | -9.52 | 114 | 2A9K | E | -10.25 |
| 51 | 1NVU | O | -7.43 | 115 | 1AY7 | O | -13.23 |
| 52 | 1JWH | E | -11.14 | 116 | 1HE8 | O | -7.37 |
| 53 | 1MAH | E | -14.51 | 117 | 1A2K | O | -9.31 |
| 54 | 1BVK | A | -10.53 | 118 | 1R6Q | E | -8.84 |
| 55 | 1BVN | E | -15.06 | 119 | 1FSK | A | -13.12 |
| 56 | 2WPT | O | -10.67 | 120 | 1F34 | E | -14.19 |
| 57 | 2GOX | O | -12.08 | 121 | 2SNI | E | -15.96 |
| 58 | 1ZM4 | E | -8.03 | 122 | 1BJ1 | A | -11.55 |
| 59 | 1P2C | A | -13.63 | 123 | 2I9B | O | -12.93 |
| 60 | 2FJU | O | -7.2 | 124 | 1IBR | O | -12.07 |
| 61 | 1EER | O | -15.59 | 125 | 1BRS | E | -17.32 |
| 62 | 1MLC | O | -9.61 | 126 | 1K5D | O | -12.77 |
| 63 | 1AK4 | O | -6.43 | 127 | 1AVX | E | -12.5 |
| 64 | 2ABZ | E | -11.67 | 128 | 1AVZ | O | -6.55 |
